# Hospital and Institutional News

**Published:** 1919-05-17

**Authors:** 


### HOSPITAL AND INSTITUTIONAL NEWS.

### THE LATE SIR E. STIRLING.

We hear with the deepest regret of the death of Sir Edward Charles Stirling, M.D., C.M.G., F.R.S., of Adelaide, South Australia. Among many prominent positions which he held with conspicuous ability were Hon. Surgeon and Lecturer on Physiology at St. George's Hospital, London, Professor of Physiology at Adelaide University; Consulting Surgeon at the Adelaide Hospital; and Director of the South Australian Museum. He was President of the Medical Congress in Adelaide in 1905. For three years he sat in the South Australian Parliament where both the power of his speeches and the clearness of his views earned him a reputation second only to that derived from his scientific ability. As an old friend states, Adelaide owes him a debt of gratitude for long years of arduous and successful work in connection with the University, Medical School, and Museum, and the students in his classes a deeper one for the noble example he set them. His work will live after him and his place will be more than difficult to fill.

### THE PROPOSED AMERICAN HOSPITAL.

Mr. Philip Franklin, F.R.C.S., the secretary of the proposed American Hospital in London, is likely to see his pet scheme fulfilled. A formal start is about to be made, and the medical staff is virtually decided on. Mr. Choyce, Dr. J. Eyre, and Dr. Eric Pritchard are among its members. American medical men will form the house staff, and the honorary medical committee will include eminent practitioners of both nations. Any plan to perpetuate the new links formed between the United States and ourselves is welcome, and nothing could be happier than a joint scheme for an American hospital in London on carefully thought-out lines.

### RETURN OF INFECTIOUS SOLDIERS.

A recent article appearing in the *Times* asserts at the outset that, "it is quite clear that too little attention is being paid to the discharge of men from the Army who may be the victims of infectious diseases and so may spread these diseases among the civil population." Now this is a somewhat sweeping statement to make after a period of some months, in which the medical journals have been full of accounts of preventive measures past, present, and in process of development. We at once admit the possibility of the entry of all manner of infectious diseases and have, in fact, often made mention of it in these columns, but we find it hard to believe that all the recent activity in such matters can be but smoke without a flame. Surely a wealth of literature and discussions such as produced at the recent B.M.A. clinical meeting has not failed to reach medical men in every branch of the profession. Later are mentioned the War Office and the Pensions Ministry. We hold no brief for the efficiency of either, but with regard to the latter, it falls to the lot of the author of this note to perform sundry laboratory investigations at the request of its officials, and this upon soldiers returned from the War zones. The impression gained is that the medical officers concerned are intent upon the most exact elucidation of every mystery in the cases they review, and that this attitude is of by no means of recent origin. The menace of disease entry to this country is certainly great and inevitable, and the organisation completely to prevent it must be ideal; but we feel that attention and endeavour are by no means wanting in our first line of defence—the younger men of the R.A.M.C. and the Ministry of Pensions' Board.

### THE PEOPLE'S LEAGUE OF HEALTH.

A meeting of the Medical Council of the "People's League of Health" was held at the Savoy Hotel, London. Major-General Sir Robert Jones, C.B., F.R.C.S., was voted to the Chair. Miss Olga Nethersole, Honorary Organiser of the Movement, read a statement of the action she had taken to bring the objects of the League to the notice of various Medical, Official, and Administrative authorities throughout the Kingdom. After a general discussion it was decided to recommend to the General Committee the establishment of a "People's League of Health," the objects of which are: "The raising of the standard of health of the British Empire. To inquire into the social problems which necessarily govern the health of the nation, and to use its strength and means to improve the social and economic conditions of the masses. To disseminate knowledge on health—how to obtain and preserve it." It was further resolved that branches of the League be formed in all cities and towns throughout the United Kingdom with a population of more than 20,000. It was decided that the General Committee should proceed upon the lines suggested in the scheme submitted by Miss Nethersole and that, with the object of placing it upon a working basis, the General Committee should in the meantime get into direct touch with the Y.M.C.A., the Y.W.C.A., the Salvation Army, and the Church Army. The Medical Council intimated its willingness to go into all matters of detail so soon as the General Committee made the necessary recommendations. A joint meeting between representatives of the General Committee and the Medical Council will be held at an early date.

### CRIPPLED CHILDREN'S HOSPITAL EXTENSION.

Those who recognise the scientific importance and the social value of the work being done at the Lord Mayor Treloar Crippled Children's Hospital, at Alton, Hampshire, in the treatment of surgical tuberculosis, will read with satisfaction of the latest development of that work in the opening of a branch seaside hospital at Hayling Island, Hampshire. A long waiting-list had brought to the notice of the Trustees the urgent necessity for an extension of the Hospital, and, apart from a scheme for the rebuilding of the present establishment, they had had under consideration for the past two or three years the desirability of establishing a seaside branch of the Hospital. Dr. Gauvain, the medical superintendent at Alton, during several summers spent some time investigating along the South Coast for a suitable site, and eventually decided on one at Hayling Island. An estate has been purchased there, and the Hospital is being rapidly completed. It is expected to have 50 patients under treatment there by mid-summer. It should be understood that the Hayling Island establishment is a branch hospital and not simply a convalescent home. The treatment given will be under the personal supervision of Dr. Gauvain and will be along the lines which have been so successful at Alton. As a "follow-up" of the good work done at Alton, a Convalescent Home has been opened at Wadhurst, to which it is proposed to send ex-patients who may require for a time convalescent treatment as an assistance to complete sure, or to prevent a relapse in cases where this threatens.

### OPEN-AIR SCHOOL SHORTAGE.

In consideration of the problems dealing with juvenile welfare, very close and disinterested co-operation between the local health and education authorities is necessary, and work in London of the medical inspection and treatment of children attending elementary schools has revealed in a remarkable way, the possibilities in this direction. The value of the work, an essential part of which has been largely carried out by voluntary help, cannot be over-estimated. The clause in the new Education Act conferring similar powers on the Higher Education Authorities bears witness to public appreciation of its efficacy, and it is interesting to note that the Cheshire Education Authority is losing no time in availing itself of its new power in the secondary schools. Much remains to be done, however, to ensure that time, money and skill are not wasted through lack of adequate preventive and remedial institutions, and in this connection a need which at once occurs to the Londoner is the establishment of many more open-air schools. In ordinary elementary schools, through the comprehensive scheme of medical examination, the children who would reap permanent benefit from attendance at such special schools are discovered. The numbers are large, but, owing to the present totally inadequate accommodation, pitifully few gain admittance, and these only remain for a year or so, many returning to the ordinary school. To any one viewing the matter from an economic point of view alone, it is surely a sounder investment to place these children when young in healthy air under medical supervision, educating them for out-of-door occupations suited to their condition, and thus equipping them mentally and physically as self-supporting citizens, than to maintain a *laissez faire* attitude until later in life they have to be supported in sanatoria. It is well known that the early stage of tuberculosis in a child yields very readily to the right treatment, and it is, therefore, well worth while to turn our attention to the delicate child, the child with tubercular tendency and with the phthisical family history. Establishment of many more day schools on the lines of the existing Birley House Open-Air School would meet an urgent need and go far towards helping in the campaign against consumption.

### STILL-BORN CHILDREN.

In the course of a recent inquiry Dr. F. J. Waldo, the City Coroner, condemned the existing system of certificates of still-born children. It was explained that a midwife could give a *declaration* of still-birth and send it to the L.C.C. This was, strictly speaking, only for the purpose of notification and not for burial, and although a midwife could be prosecuted for giving a *certificate*, there was no doubt that children were often buried on the evidence of the *declaration*, for in practice there was not much difference. The midwife's declaration was not, however, a statutory one made before a J.P. or a Commissioner of Oaths. A registered medical practitioner might declare on a piece of paper that a newly-born infant was still-born, whether he was present at the confinement or not, and a certified midwife might do the same if she was present. There was then no registration by the local registrar on reference to the coroner. The present procedure opened the door to all manner of crimes, and the lack of safeguards to the public and helpless infants ought at once to be remedied.

### HOSPITAL BEDS FOR BABIES.

The *Medical Officer* for May 3 publishes an interesting extract on the infant-welfare scheme at Manchester. An important feature is the provision of beds at the Babies' Hospital, Levenshulme, and at the Monsall Hospital. Three years ago, eighteen beds were allocated to the corporation in the Babies' Hospital under special conditions, determined partly by the fact that the source of admissions had largely been from the Child Welfare Centres of the School for Mothers. This school is medically staffed by lady practitioners and only children under one year of age are admitted, who are to be suffering from wasting, malnutrition, etc., Dr. Riven has pointed out that the cases admitted are precisely those which baffle the medical officers of the child welfare centres, and form the subject of long and inconclusive reports from the health visitors. The infant is not kept for a very long period in the hospital owing to the pressure of waiting cases; only sufficiently long to place it on the upward grade if that can be effected, and to determine the best mode of dealing with its condition. After the return of the children to their homes, observation is maintained by the health visitors and also by the hospital itself. A different policy has been pursued at Monsall Hospital. Here no limit has hitherto been placed on the stay of these children, and the results are better, at all events for the time being.

### A TYPICAL INSTANCE.

Recently our attention was drawn to the following example which apparently escaped report by the Press. Dr. Waldo tells us that he concluded on the 26th ult. after three sittings an inquest concerning the death of the illegitimate newly-born baby of a barmaid said to be the wife of a soldier. An attempt had been made to get the body buried by an undertaker as a still-born by means of a forged certificate falsely bearing the name of the midwife who had attended the mother at the Old Duke's Head, Bermondsey, and later £10 was unsuccessfully offered to the midwife to write out a certificate. Dr. Spilsbury, who made the autopsy, could not explain a mark round the child's neck, which, he said, might have been produced by a cord either before or after death. He found, however, a patent ductus arteriosus between the aorta and the pulmonary artery, which congenital defect was, he said, sufficient to produce death by suffocation. The jury returned an open verdict—death by suffocation, but by what means insufficient evidence to say. On the advice of the coroner, they added the following rider, viz., "We are strongly of the opinion that still-births which have reached the stage of seven months development should be registered upon the certificate of a registered medical practitioner; and that it should not be permitted to bury or otherwise dispose of a still-birth until an order for burial has been issued by the Registrar with this object in view. We suggest that the Home Secretary bring in a Bill to amend the Registration of Births and Deaths Act of 1874." Dr. Waldo explained that the Coroners' Society, the B.M.A., and the Select Committee on Death Certification had one and all petitioned Parliament for registration of still-births.

### £1,000 REWARD.

A lady living at Chudleigh, Devon, has written to the Royal Hospital for Incurables, Putney, offering to that institution a thousand pounds as a thank-offering if the secretary will secure for her a suitable house in a pleasant locality near London. In one sense the point of the offer will be missed if it is regarded as extraordinary; it is rather a grave sign of the times. For we have already observed advertisements in a local newspaper in Sussex which offered "three pounds reward" to any one who could tell the advertiser of a vacant house in the adjoining little town. The remarkable thing was that the offer was not hedged with conditional clauses in regard to size, suitability, and the like. The scarcity of houses has made a house at any price an offer to be considered. But the Devonshire lady is wise in her knowledge that an important institution has sources of information which are not open to private persons, and perhaps, on occasion, beyond the range of agents and commercial offices. If the Royal Hospital for Incurables gains the reward it will certainly have earned it.

### WEST LONDON HOSPITAL BENEFACTOR.

Dramatic but none the less pleasing and welcome was the announcement made by Mr. D. Mason, of Chiswick, at a gathering called in the interests of West London Hospital, Hammersmith. Mr. C. F. Marshall, the Chairman, had explained the urgent need of enlarging the premises in view of the fact that the waiting-list for the in-patients' side averaged 470, and that the demand on the out-patients' department was causing serious delay, when Mr. Mason, who is a vice-president of the hospital, rose and stated that he would provide a new out-patients' department at a cost of at least £22,000 "in gratitude for what our boys have done in the War." He gives twice who gives quickly. The Committee's scheme for the enlargement of the hospital will, it may be added, involve an expenditure of £100,000.

### A NEW SKIN HOSPITAL NEEDED.

The Birmingham Hospital for Skin and Urinary diseases has come structurally to the parting of the ways. The staff is convinced that a new hospital is desirable, and that at least extension is a necessity at once. As Dr. Felix Vinrace has put it: "The hospital is full from the roof to the cellars." Private wards and a new dispensary appear to be the most obvious needs, but the several departments are cramped, and the difficulties of the staff, and so of the patients, obtrude themselves in many ways. The way to better things has been pointed by the hospital's President, Mr. Edward Ansell, who has promised £200 towards the cost of extension. But a larger building, which will increase maintenance expenses, should not be left to the generosity of one or even of a few. Recent public enactments have opened new fields of hospital work, especially perhaps for special hospitals. The public, which will know the benefit when the new departments have been created, must help now to set them on their feet.

### RECONSTRUCTION OF WEST HERTS HOSPITAL.

After forty-two years' work, the buildings of West Herts Hospital need reconstruction, which has become the more necessary since the war postponed the scheme prepared in 1914. The whole scheme depends on the extent of the support received, but if the appeal is successful the following changes will be begun: The quarters of the nurses need improvement and extension. The children's ward is too small and out-of-date. A new $x$-ray department is required, and an electric installation for all purposes is needed. Once a new children's ward has been built the present one can be adapted into an open-air ward which has long been wanted. It is suggested that the new children's ward should be a Peace memorial, but we hope that a more ambitious memorial scheme will be adopted, so that the complete reconstruction can be carried out. Peace deserves more than a new ward to celebrate it, and West Herts will wish its memorial scheme to be worthy of the occasion, and of the needs of the hour.

### A "HORRIBLE" RUMOUR.

There has been referred to a special meeting of the Barry Hospital Committee the rumour that "horrible" things have happened at the Town Accident Hospital and the correspondence between the clerk and a medical man arising therefrom. The allegations state that an operation was performed on a patient without an anæsthetic, that she was held down during its progress, and that her cries were heard throughout the ward in which the operation is said to have taken place. The Councillor who made these statements affirmed that he had heard that the operation was performed without anæsthesia because the operator arrived after the anæsthetist had left. At the meeting at which these statements were made, a letter from the solicitor of the medical man was read. It asked for a public inquiry. The doctor, in a newspaper interview, has denied the allegations.

### TWO MEDICAL RESIGNATIONS.

THE Dreadnought Seamen's Hospital loses by resignation two of the most prominent members of its Honorary Medical Staff, Mr. William Turner, M.S., F.R.C.S., the senior surgeon, and Sir Malcolm Morris, K.C.V.O., surgeon for diseases of the skin. Mr. Turner joined the Society as long ago as 1896 when the staff was but a small one. He has seen the rise of the hospital to its present important position in the medical world, and for that great advance the Seamen's Hospital Society is in a great measure indebted to their senior surgeon. He retires in consequence of increased calls on his time, as he is now surgeon in charge of in-patients at Westminster Hospital. Sir Malcolm Morris first took office in the year 1905 on the founding of the London School of Clinical Medicine; and he has worked indefatigably on behalf of both the hospital and school since that date. In recognition of the services which they have rendered the Board of Management have elected both of them members of the consulting staff.

### BY BOAT TO HOSPITAL.

EAST ANGLIA is still somewhat a world to itself, with its flats, its marsh, and its rivers. Consequently, when the great appeal on behalf of the East Suffolk and Ipswich Hospital is met by a small response from those districts, like Harwich, which have immature schemes for future cottage hospitals of their own, it refers not only to the two ambulance cars that are to serve the remoter districts, but also to the revival of the river-boat service. The Great Eastern Railway has promised not only to revive and improve the water transit between Ipswich and Harwich, Felixstowe and Dovercourt, but the service itself has been preferred to the roundabout railway by the residents in the places named. Whether the local funds will be merged in the central appeal fund is another matter. But if they are not, the localities will have to show greater advances in their plans than, till the Ipswich Hospital took the field, seemed likely. Mr. A. Griffiths is an experienced shepherd, and we have no doubt that he will bring all the sheep to the fold by satisfying them that their interests are as safe in his hands as in their own. It is a great moment for the Ipswich Hospital.

### IN THE WAKE OF THE MAINS.

THE rural district of Kirbymoorside in Yorkshire is to have a cottage hospital, if the lead of Mrs. Edward Shaw and her friends is to be followed. The idea is to found a small institution in Welburn Parish, near Howkeld; but the plan depends on the extension of the water main from Starfits Lane to Howkeld. Mrs. Shaw has offered to lend to the rural district council the necessary funds below 6 per cent., the rate for certain public loans. Thanks to her public spirit and the generosity of her sister and two others the council is ready to proceed, and the scheme is likely to be completed.

### DISPENSERS AT INSTITUTIONS.

AFTER a period of eleven years since the passing of the "Poisons and Pharmacy Act, 1908," the Pharmaceutical Society propose to take action under Section 4 (B) of that Act and admit to the statutory Register of Chemists and Druggists persons who have held the "Assistant's" Certificate in Dispensing of the Society of Apothecaries. Briefly, the conditions require that applicants for registration shall have held the Assistant's Certificate *before December 1908* and shall have been for seven years immediately prior to their application "in charge" of the dispensing at a hospital or institution approved by the Council of the Pharmaceutical Society. Applicants who are accepted must pay the registration fees of 14 guineas, as those do who qualify by examination. Opposition is likely to come from the general body of Pharmacists, who resent the admission to the Register of persons whose chief claim is that they have been in charge of a dispensary for seven years and hold the Assistant's Certificate in Compounding,—this, it should be explained, may be obtained after six months' study, as opposed to the three years' curriculum required for the statutory qualification of Pharmacist. The President of the Pharmaceutical Society explains that it was on account of pressure from the Privy Council that these steps had been taken; in the opinion of the Pharmaceutical Council, the clause in the Act was "permissive" only. The by-law has to be read three times at meetings of the Council and then requires the sanction of members at a general meeting.

### THIS WEEK'S DRUG MARKET.

THERE is more activity in the market, and the downward tendency of values is neither so pronounced nor so general as it has been for the past few months. With the exception of many, perhaps most, of the synthetic drugs which still move downwards in value, it is possible that the recession of prices will be slower now that purchasers at last appear to have decided to come on to the market. At the recent public sale of drugs in Mincing Lane the demand was much better than it has been for at least two or three years, and a fair proportion of the offerings found buyers in some cases at higher rates. Cardamoms and rhubarb are dearer. A good business has been done in gum benzoin at fully maintained rates. Even honey has a firmer price tendency. Quicksilver is about 7½ per cent. dearer, and the prices of calomel, corrosive sublimate, and other salts of mercury may be advanced in proportion. English refined camphor is dearer. Ergot of rye is still scarce and very high prices are wanted. Values of menthol and peppermint oil are firmly maintained. Lemon oil has a lower price tendency, and the same may be said of cocaine, barbitone, and aspirin.

